# Synergistic carcinogenesis of the nasopharyngeal microbiome and Epstein-Barr virus: mechanisms of metabolic reprogramming and immune evasion

**DOI:** 10.3389/fimmu.2026.1771414

**Published:** 2026-02-17

**Authors:** Shasha Shen, Juan Li, Sijia Zheng, Xue Cui, Xiaoxia Gou

**Affiliations:** Head and Neck Oncology, The Second Affiliated Hospital of Zunyi Medical University, Zunyi, China

**Keywords:** bacteriophage therapy, Epstein-Barr virus (EBV), immune evasion, metabolic paradox, microbiome, nasopharyngeal carcinoma (NPC), organoid models, synergistic carcinogenesis

## Abstract

Nasopharyngeal carcinoma (NPC) is strongly associated with Epstein-Barr virus (EBV) infection, but EBV alone is insufficient for tumorigenesis. Recent evidence suggests that the nasopharyngeal microbiome plays a critical, yet underexplored, role in NPC development. This review investigates the synergistic interaction between EBV and the nasopharyngeal microbiome, focusing on microbial dysbiosis and its role in NPC pathogenesis. We highlight significant microbial dysbiosis in NPC patients, characterized by an overgrowth of opportunistic pathogens such as *Fusobacterium nucleatum* and *Porphyromonas gingivalis*. These pathogens interact with EBV-infected epithelial cells, amplifying oncogenic signaling through the NF-κB and PI3K/AKT pathways. Crucially, we explore the “SCFA paradox,” where microbial short-chain fatty acids (SCFAs), typically beneficial, act as HDAC inhibitors that paradoxically trigger EBV lytic reactivation in B-cells. Additionally, the microbiome facilitates immune evasion through interactions between *F. nucleatum* Fap2 and the TIGIT receptor, in synergy with EBV’s LMP1 protein. These findings underscore the importance of the microbiome in NPC pathogenesis and highlight the potential for integrating microbial signatures into diagnostic tools. We conclude by discussing precision therapies, such as bacteriophage treatment, and emphasize the role of next-generation models—specifically Air–Liquid Interface organoids—as functional ‘patient avatars.’ These systems are essential for advancing personalized medicine, as they enable the functional validation of individualized microbial interventions that sequencing alone cannot predict.

## Graphical Abstract

**Figure:**
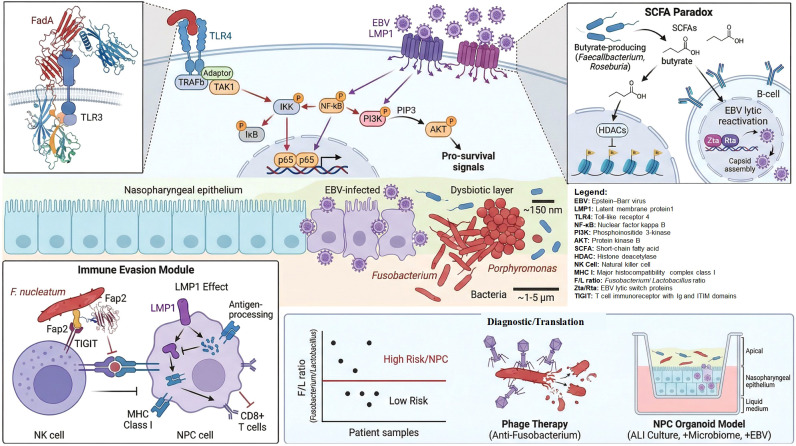

## 1 Introduction

Nasopharyngeal carcinoma (NPC) is a distinct malignancy that primarily affects individuals in endemic regions such as Southern China, Southeast Asia, and North Africa (1). The etiology of NPC is inextricably linked to the Epstein-Barr virus (EBV), which is present in nearly all cases of undifferentiated non-keratinizing NPC (2). Despite the pervasive nature of EBV, only a small subset of infected individuals develop NPC. This epidemiological paradox implies that EBV infection is necessary but insufficient for tumorigenesis, suggesting that additional cofactors within the unique nasopharyngeal microenvironment are required (3, 4).

Emerging evidence has highlighted the nasopharyngeal microbiome as a critical, yet historically overlooked, driver in this niche. Unlike the sterile environment assumed in early pathology, the nasopharynx functions as a unique aerobic-anaerobic transition zone within Waldeyer’s ring (5, 6). Recent studies reveal that NPC patients exhibit profound dysbiosis, characterized by the enrichment of opportunistic pathogens—specifically *Fusobacterium nucleatum* and *Porphyromonas*—directly within the tumor tissue (7–9). This dysbiosis is not merely a bystander effect; it actively promotes EBV reactivation and amplifies oncogenic signaling (e.g., NF-κB and PI3K/AKT) (10).

Our review aims to elucidate the synergistic mechanisms between the nasopharyngeal microbiome and EBV, moving beyond general associations. Crucially, we critically evaluate the “SCFA Paradox”: while microbial short-chain fatty acids (SCFAs) like butyrate are beneficial in the gut, they function as histone deacetylase (HDAC) inhibitors in the nasopharynx, paradoxically triggering EBV lytic reactivation in the underlying lymphoid tissue (11–13). Furthermore, we explore how the nasopharyngeal microbiome aids in collaborative immune evasion, particularly through the interaction between the bacterial adhesin Fap2 and the inhibitory receptor TIGIT (14).

By integrating these insights, we propose that the nasopharyngeal microbiome serves as a vital target for precision medicine. We discuss the potential of novel diagnostic markers, such as the Fusobacterium-to-Lactobacillus (F/L) ratio (15), and advocate for targeted interventions like bacteriophage therapy to dismantle this pro-tumorigenic synergy (16, 17).

## 2 The unique microbial landscape of NPC

### 2.1 The nasopharynx: a distinct ecological niche

The nasopharynx represents a unique anatomical and ecological interface that differs significantly from the well-studied oral and gastrointestinal tracts. Unlike the oral cavity, which is constantly flushed by saliva and subject to rapid pH fluctuations, or the gut, which is a predominantly anaerobic, nutrient-rich lumen, the nasopharynx functions as a distinct aerobic-anaerobic transition zone (5). Connecting the nasal cavity to the oropharynx, it maintains a gradient of oxygen tension that allows for the co-existence of aerobic commensals and facultative anaerobes. This specific microenvironment serves as the primary reservoir for EBV transmission and persistence, acting not merely as a passive airway but as an active immune interface where the host epithelium, resident microbiota, and latent viral reservoirs intersect (18, 19). Crucially, the unique mucociliary clearance and immune surveillance mechanisms in this region imply that microbial colonization requires specific adaptive strategies, such as biofilm formation, which may shelter pathogens and facilitate chronic interactions with, in the case of infection, the underlying EBV-infected epithelium (20, 21).

**Figure 1:**
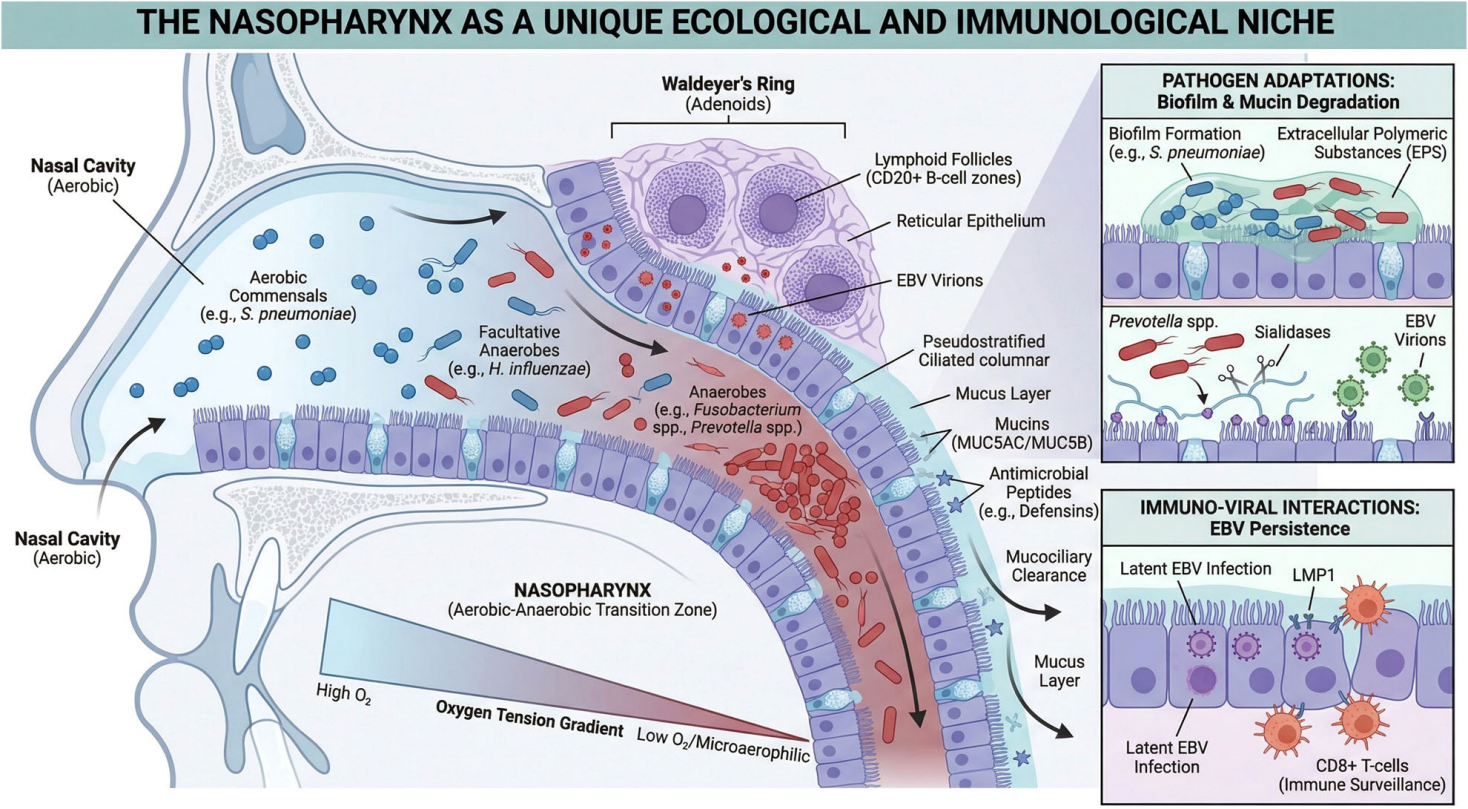
The nasopharynx as a unique ecological and immunological niche: architectural and microbial features driving EBV persistence. The main illustration, Ecological and Anatomical Features, shows the nasopharynx as a distinct Aerobic-Anaerobic Transition Zone. The top right inset, Pathogen Adaptations: Biofilm & Mucin Degradation, illustrates how dysbiotic pathogens compromise mucosal defenses. The bottom right inset, Immuno-Viral Interactions: EBV Persistence, details how latent EBV persists within the lymphoid stroma despite immune surveillance.

Furthermore, the nasopharynx constitutes a central component of Waldeyer’s ring, a ring of lymphoid tissue that includes the adenoids and tonsils. This unique “lymphoepithelial” architecture facilitates intimate contact between surface-colonizing microbes, the overlying reticular epithelium, and the abundant underlying lymphoid infiltrate (the primary reservoir of latent EBV) (6). Unlike the keratinized squamous epithelium of the skin, the nasopharyngeal mucosa is overlaid by a mucus layer rich in mucins (e.g., MUC5AC, MUC5B) and antimicrobial peptides (22). In a healthy state, this barrier entraps bacteria and facilitates their removal via mucociliary clearance. However, dysbiotic pathogens often evolve mechanisms to breach this defense; for instance, certain anaerobes secrete mucin-degrading enzymes (e.g., sialidases and glycosylases), effectively dissolving the protective layer and exposing the vulnerable epithelium to direct viral-bacterial contact (23). This unique anatomical and ecological relationship facilitates the microbial breach of the mucosal barrier and the establishment of the oncogenic niche, as visually summarized in **Figure 1**.

### 2.2 Microbial dysbiosis in NPC: shifting from commensalism to pathogenicity

In the progression from healthy nasopharyngeal mucosa to carcinoma, the microbial landscape undergoes a profound shift, characterized by a collapse of homeostasis and the emergence of a pro-tumorigenic profile (24).

#### 2.2.1 Reduced diversity and resilience

Consistent with findings in other mucosal malignancies, NPC is marked by a significant reduction in alpha diversity (25). The stable ecosystem seen in healthy individuals—typically dominated by Proteobacteria and Firmicutes—collapses into a dysbiotic state (5, 24). This loss of microbial complexity often indicates a breakdown in “colonization resistance,” rendering the niche susceptible to invasion by opportunistic pathogens (26).

#### 2.2.2 Enrichment of opportunistic pathogens

A hallmark of the NPC microbiome is the specific enrichment of anaerobic, periodontal-associated pathogens (8, 24). Groundbreaking recent studies utilizing spatial multi-omics and high-depth sequencing have provided direct tissue-level evidence of this phenomenon (7). Notably, Tan et al. (2025) demonstrated that *Porphyromonas gingivalis* and *Fusobacterium nucleatum* are not merely present but are spatially co-localized with EBV-infected tumor cells within the tissue microenvironment (9). These findings suggest a direct physical interaction rather than a distant systemic effect. Similarly, recent spatial and tissue-level studies provide direct evidence for microbial colonization. For example, Liao et al. (2024) reported that oral microbes translocate to the nasopharynx and infiltrate tumor tissues, correlating with EBV infection (8). Consistent with this, prior lavage-based 16S studies (24) and recent dysbiosis analyses demonstrate a structural collapse of the nasopharyngeal microbiome, characterized by reduced diversity and the enrichment of opportunistic pathogens. The enrichment of these anaerobes in the nasopharynx suggests they may exploit the hypoxic conditions generated by the rapidly growing tumor, creating a feed-forward loop of inflammation and viral reactivation.

#### 2.2.3 Loss of beneficial commensals

Concurrently with pathogen enrichment, there is a marked depletion of health-associated genera, particularly *Lactobacillus* and *Bifidobacterium (*27). In a healthy state, these commensals contribute to mucosal barrier integrity and produce anti-inflammatory metabolites that maintain immune quiescence. Their depletion in NPC patients removes a critical “brake” on inflammation, potentially creating a permissive environment for EBV-driven oncogenesis (8). To provide a comprehensive overview of this shifting microbial landscape, we summarize key recent investigations and mechanistic insights linking specific nasopharyngeal taxa to EBV kinetics and NPC pathogenesis in **Table 1**. However, the findings presented in this table should be interpreted with academic caution, as the nasopharynx is a dynamic environment with high bacterial turnover, and microbial signatures may vary significantly across different sample types, geographical regions, and population-specific cohorts.

**Table 1:** Key recent studies investigating the microbiome-EBV nexus in nasopharyngeal carcinoma (2020–2025).

Study (author, year)	Sample type	Key bacterial signatures	Major findings & mechanisms
Xia et al. (2021) (27)	Clinical Data & Animal Model	Depletion: *Lactobacillus*, *Bifidobacterium*	Loss of Mucosal Barrier: Depletion of these beneficial genera reduces anti-inflammatory metabolites, removing the “brake” on inflammation and compromising barrier integrity.
Wang et al. (2024) (8)	Nasopharyngeal Swab	Enrichment: *Fusobacterium nucleatum*, *Prevotella*	Synergistic Activation: Confirmed oral-to-nasopharyngeal translocation. *F. nucleatum* load correlates with EBV DNA and upregulates LMP1 expression via NF-κB pathway activation.
Zhang et al. (2023) (10)	Saliva & Swab	Enrichment: *Streptococcus sanguinis*	Metabolic Reactivation: Identified that this bacterium produces Hydrogen Peroxide (H_2_O_2_), acting as a metabolic switch to induce EBV lytic reactivation and genomic instability.
Tan et al. (2025) (9)	Biopsy Tissue (Spatial Multi-omics)	Enrichment: *Fusobacterium nucleatum*, *Porphyromonas gingivalis*	Direct Co-localization: Provided first *in situ* evidence of periodontal pathogens physically co-localizing with EBV-infected tumor cells, suggesting direct micro-environmental interaction.

The findings summarized in **Table 1** provide a compelling overview of microbial shifts, yet they must be interpreted with academic caution. The nasopharynx is a dynamic environment with high bacterial turnover, and the signatures observed in nasopharyngeal swabs may not consistently align with those found in deep biopsy tissues. Furthermore, these microbial profiles are often influenced by geography-related variables and population-specific factors (e.g., diet and environmental exposures) within the analyzed cohorts. It is also plausible that current sequencing methodologies may overlook low-abundance but biologically significant taxa, potentially leading to a biased view of the ecosystem (28, 29).

In conclusion, the transition from commensalism to pathogenicity in NPC is best understood through a “pathogen-driver/commensal-passenger” framework. In this context, specific transversally present anaerobes—such as Fusobacterium—act as “drivers” that reshape the microenvironment and facilitate EBV kinetics, while the loss of beneficial commensals like Lactobacillus acts as a “passenger” effect that further compromises mucosal homeostasis.

## 3 Mechanisms of viral-bacterial synergism

The pathogenesis of NPC is not merely the result of additive insults but rather a synergistic interplay where microbial dysbiosis and EBV infection mutually reinforce oncogenic signaling. This synergism operates through three primary axes: the co-activation of inflammatory pathways, the paradoxical regulation of viral latency by microbial metabolites, and the collaborative evasion of host immunity (8, 10, 30).

### 3.1 The “Double Hit” on signaling pathways

A critical mechanism driving NPC tumorigenesis is the convergence of bacterial and viral signals on the NF-κB and PI3K/AKT pathways, creating a “double hit” that sustains cell survival and promotes malignant transformation.

#### 3.1.1 Bacterial activation

Opportunistic pathogens enriched in the NPC niche, particularly *Fusobacterium nucleatum* and *Porphyromonas gingivalis*, possess potent virulence factors. F. nucleatum utilizes its adhesin FadA to bind E-cadherin on epithelial cells. Crucially, this interaction extends beyond structural disruption; the binding of FadA facilitates the phosphorylation and nuclear translocation of β-catenin (31, 32). Once in the nucleus, β-catenin complexes with TCF/LEF transcription factors to initiate a transcriptional program that drives cell cycle progression (e.g., via *CCND1* and *MYC*) and inflammatory cytokine production. Concurrently, lipopolysaccharides (LPS) from the outer membranes of gram-negative bacteria engage Toll-like receptors (TLR2/4), further amplifying the production of pro-inflammatory cytokines such as IL-6 and TNF-α. It is important to note that direct bacteria-cell contact is not strictly required to trigger these signaling cascades. Gram-negative pathogens like F. nucleatum continuously shed Outer Membrane Vesicles (OMVs)—nanoscale proteoliposomes enriched with virulence factors, LPS, and genetic material. These OMVs function as long-range delivery vehicles. They can diffuse through the mucus layer and fuse with the plasma membrane of epithelial cells, delivering their pro-inflammatory cargo deep within the tissue microenvironment. This mechanism allows dysbiotic bacteria to modulate host signaling, promote metastasis, and alter the immune landscape even in tumor regions that lack direct bacterial colonization, significantly expanding the “radius of influence” of the microbiome (32, 33).

#### 3.1.2 Viral amplification

In isolation, these bacterial signals might trigger acute inflammation that is eventually resolved. However, in EBV-infected cells, the viral oncoprotein LMP1 acts as a constitutively active mimic of the TNF receptor (CD40). LMP1 recruits Tumor Necrosis Factor Receptor-Associated Factors (TRAFs) and Tumor Necrosis Factor Receptor Type 1-Associated Death Domain proteins(TRADDs) to the cytoplasmic membrane, intrinsically driving the non-canonical NF-κB pathway to block apoptosis (34).

#### 3.1.3 The synergistic outcome

**Figure 2:**
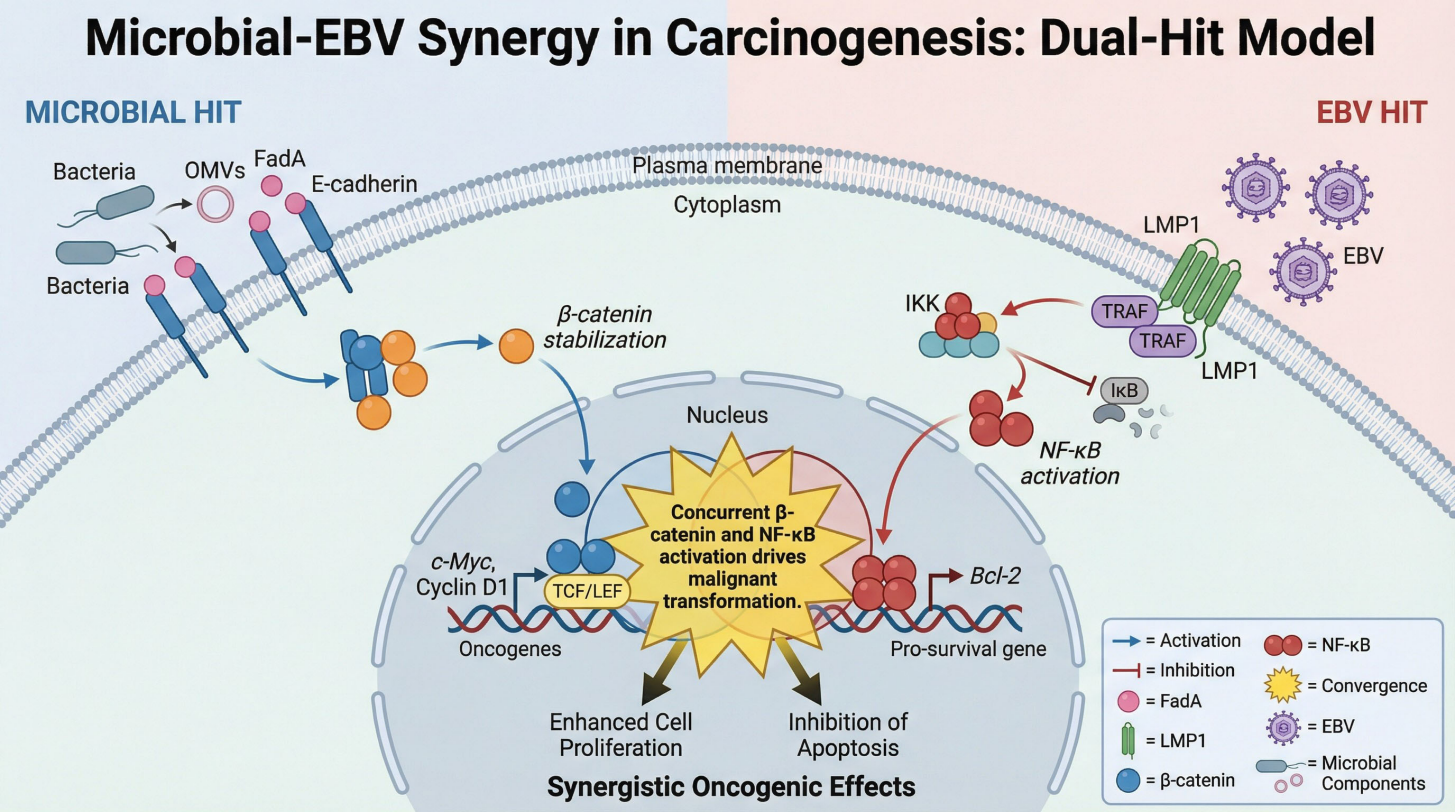
Microbial-EBV synergy in carcinogenesis: the dual-hit model driving oncogenic transformation. The Microbial Hit (left) illustrates pathogenic bacteria triggering β-catenin stabilization via FadA binding. The EBV Hit (right) shows EBV LMP1 activating the NF-kB pathway. The Synergistic Oncogenic Effects (bottom center) demonstrates how these converging signals drive oncogene expression, cell proliferation, and inhibition of apoptosis.

When dysbiotic bacteria colonize EBV-positive epithelium, the extrinsic bacterial signals (TLR/FadA) and intrinsic viral signals (LMP1) converge. This creates a feed-forward loop where inflammation upregulates LMP1 expression, and LMP1, in turn, sensitizes cells to external stimuli. The functional consequence is a profound promotion of Epithelial-Mesenchymal Transition (EMT), genomic instability via ROS production, and the acquisition of an immortalized phenotype that neither the virus nor the bacteria could achieve efficiently alone (8, 35, 36). This synergistic signaling cascade is illustrated in **Figure 2**.

### 3.2 Metabolic reprogramming and the SCFA paradox

While short-chain fatty acids (SCFAs) like butyrate are widely recognized for their anti-tumorigenic properties in colorectal cancer, their role in the EBV-associated nasopharyngeal niche represents a complex biological paradox. Commensal bacteria (e.g., butyrate-producing *Clostridia* clusters) secrete SCFAs that function as Histone Deacetylase (HDAC) inhibitors. At a molecular level, this inhibition prevents the removal of acetyl groups from histone tails, leading to a state of global hyperacetylation (particularly on H3 and H4). This epigenetic remodeling relaxes the condensed chromatin structure, rendering DNA more accessible to transcription factors (37, 38).

#### 3.2.1 Critical analysis of the paradox

- In B-cells (The Trigger): Within the lymphoid stroma surrounding the nasopharynx, butyrate-mediated HDAC inhibition relaxes the chromatin structure at the promoter of the EBV immediate-early gene *BZLF1* (ZEBRA) (13). Specifically, the accumulation of butyrate reverses the heterochromatic silencing at the *Zp* promoter, facilitating the binding of transcription factors like ZEBRA and Rta. This triggers the switch from latency to the lytic cycle, leading to the production of infectious virions. This “reactivation” is crucial for disease progression, as it facilitates the continuous infection of adjacent epithelial cells and maintains high viral loads (39).
- In Epithelial Cells: HDAC Inhibition and Cytotoxicity: Conversely, in established nasopharyngeal carcinoma cells, high concentrations of HDAC inhibitors predominantly induce cell cycle arrest and apoptosis, acting as a check on tumor growth (40). This creates a distinct contrast to their pro-viral effect in the lymphoid compartment.

#### 3.2.2 The concentration-dependency nuance

A critical consideration raised by the dysbiotic nature of NPC is the quantitative relationship between SCFA producers and their biological effects. While the transition from a homeostatic state to NPC-associated dysbiosis is characterized by a marked loss of primary commensal producers (such as Clostridiales), the “SCFA Paradox” remains functionally relevant due to two key factors (41, 42).

First, the biological impact appears to be strictly concentration-dependent; the induction of the EBV BZLF1 promoter requires a relatively low concentration threshold (typically 0.5–2 mM) compared to the higher, sustained concentrations necessary for direct epithelial cytotoxicity. Second, certain opportunistic pathogens enriched in the NPC niche, such as Fusobacterium and Porphyromonas, are capable of producing significant levels of butyrate and acetate as metabolic byproducts of protein fermentation (43, 44).This suggests that even a diminished proportion of SCFA producers in a dysbiotic environment (e.g., 10% *vs*. 40% in healthy states) can maintain localized “hotspots” of metabolites within the restricted nasopharyngeal crypts that are sufficient to trigger viral reactivation (45, 46).

#### 3.2.3 Conclusion of the section

This dichotomy suggests that microbial metabolites act as a “double-edged sword.” In the pre-malignant nasopharynx, a “healthy” or pathologically altered SCFA-rich microbiome might inadvertently fuel viral dissemination from the B-cell reservoir, whereas in established tumors, it might exert direct cytotoxic effects. Understanding this context-dependent response is vital for evaluating the safety of probiotic interventions (13). Thus, the specific microenvironment of the nasopharynx dictates that metabolites beneficial in the gut may act as ‘oncometabolites’ or viral triggers in NPC, challenging the ‘one-size-fits-all’ approach of probiotic therapy (47, 48).

#### 3.2.4 Beyond SCFAs: the nitrate-nitrite-nitrosamine axis

Beyond the complex role of SCFAs, the metabolic contribution of the microbiome extends to the processing of dietary carcinogens. Epidemiological studies have long established a robust link between NPC and the consumption of preserved foods rich in N-nitrosamines (e.g., Canton-style salted fish) (1, 49, 50). While traditional Cantonese-style salted fish is a well-documented risk factor in endemic regions such as Southern China, N-nitrosamines and their precursors are also prevalent in globally consumed processed meats (e.g., bacon, sausages, and deli meats) and certain pickled vegetables (51).

It should be clarified that while the most potent epidemiological links are observed in specific populations with lifelong traditional dietary habits, the microbial conversion of these dietary nitrates into pro-carcinogenic nitrosamines represents a transversal mechanism relevant to broader populations consuming highly processed diets (52). Crucially, the reduction of dietary nitrate to nitrite—a prerequisite for endogenous nitrosamine formation—is performed almost exclusively by oral and nasopharyngeal bacteria (e.g., specific strains of *Veillonella* and *Actinomyces*) possessing nitrate reductase activity (53, 54).

In the dysbiotic nasopharynx, an overgrowth of these nitrate-reducing bacteria acts as a metabolic “catalytic converter.” They amplify the local concentration of carcinogenic N-nitroso compounds, which directly induce DNA alkylation and mutagenesis in the adjacent epithelium. This highlights a potentially overlooked “Diet-Microbiome-Oncogenesis” axis, where the microbiome dictates the genotoxic potency of dietary precursors (55, 56). To synthesize the dense mechanistic details presented throughout this section, **Table 2** provides a comprehensive reference, detailing the specific microbial components, their host or viral targets, and the resulting oncogenic outcomes.

**Table 2:** Comparative analysis of SCFA roles in gut *vs*. nasopharyngeal niches.

Metabolic axis	Key microbial players	Host/viral target	Molecular mechanism	Oncogenic outcome
The “SCFA Paradox”	*Porphyromonas*, *Fusobacterium*, *Clostridia* spp.	EBV *BZLF1* (Zp) Promoter (in B-cells)	HDAC Inhibition: Butyrate inhibits Histone Deacetylases, causing H3/H4 hyperacetylation and chromatin relaxation at the *Zp* promoter.	Lytic Reactivation: Triggers the switch from latency to the lytic cycle, fueling viral dissemination to the epithelium.
Nitrate-Nitrite Axis	*Veillonella*, *Actinomyces* spp.	Dietary Nitrate/Host DNA	Enzymatic Reduction: Nitrate reductases convert dietary nitrate into nitrite, facilitating endogenous N-nitrosamine formation.	Chemical Mutagenesis: Elevates local concentrations of N-nitroso compounds, causing DNA alkylation and mutations.

### 3.3 Collaborative immune evasion

Immune evasion is a hallmark of NPC, and emerging data suggest this is a coordinated effort between the virus and the dysbiotic microbiome.

#### 3.3.1 Bacterial contributions

*Fusobacterium nucleatum* is a potent immune modulator. It recruits Myeloid-Derived Suppressor Cells (MDSCs) and regulatory T cells (Tregs) to the tumor site, creating an immunosuppressive shield. Furthermore, it has been shown to inhibit the cytotoxicity of NK cells and CD8+ T cells via direct receptor-ligand interactions. Recent structural biology studies suggest that the fusobacterial lectin Fap2 binds directly to the inhibitory receptor TIGIT expressed on NK cells and T cells. This binding mimics the “self-recognition” signal, effectively applying an immunological brake that prevents the clearance of malignant cells (14).

#### 3.3.2 Viral contributions

Simultaneously, EBV-encoded LMP1 downregulates MHC Class I molecules and antigen-processing machinery (TAP), effectively “hiding” the infected cells from cytotoxic T-lymphocyte recognition (57, 58).

#### 3.3.3 The immunosuppressive TME

**Figure 3:**
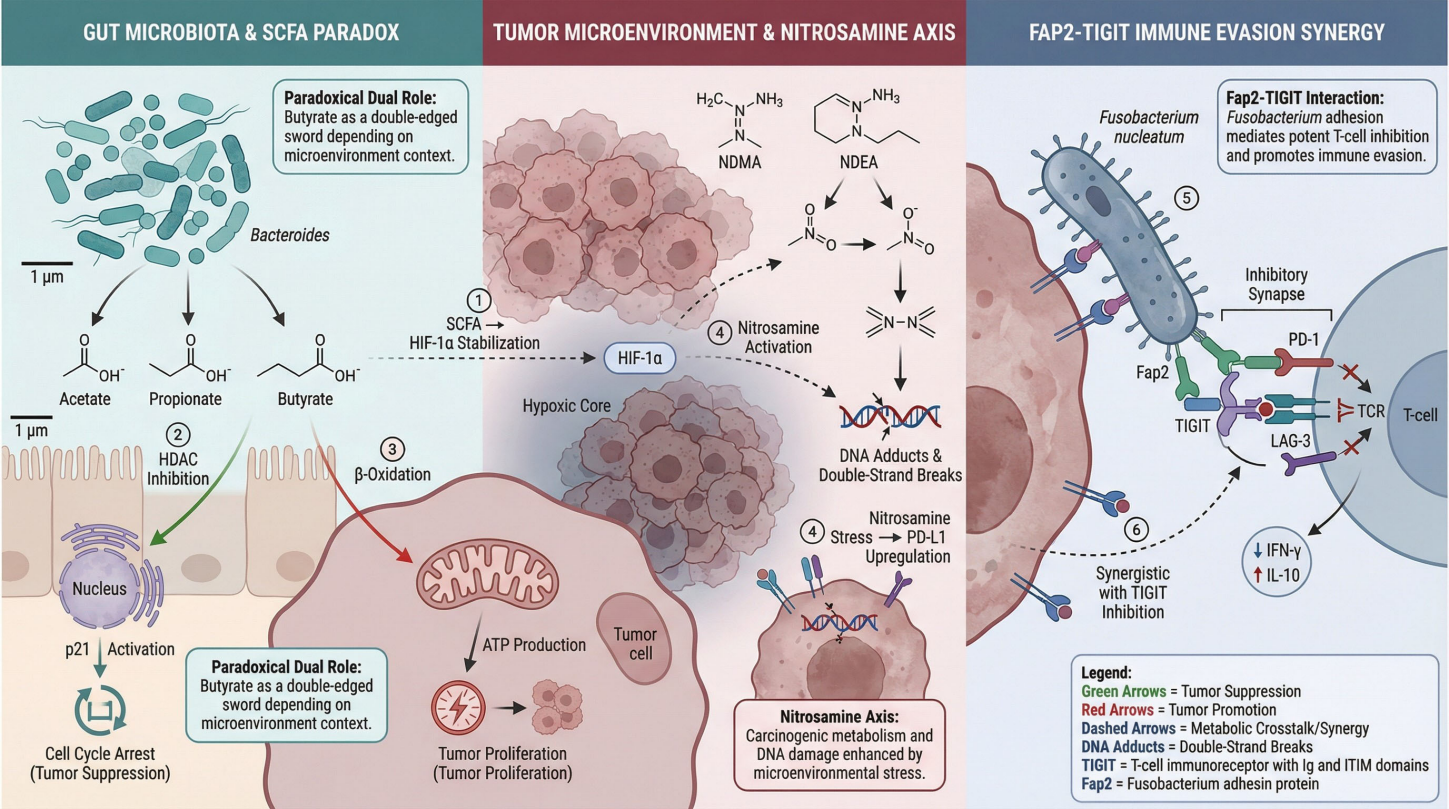
The complex metabolic microenvironment and collaborative immune evasion in NPC: the SCFA paradox and Fap2-TIGIT synergy. The Gut Microbiota & SCFA Paradox panel (left) contrasts the dual roles of butyrate. The Tumor Microenvironment & Nitrosamine Axis panel (middle) shows the activation of carcinogenic precursors. The Fap2-TIGIT Immune Evasion Synergy panel (right) illustrates the direct microbial contribution to T-cell inhibition and tumor evasion.

Together, these mechanisms construct a “cold” tumor microenvironment (TME). The bacteria prevent immune cells from infiltrating or functioning within the tumor (via checkpoint inhibition), while the virus minimizes the antigenicity of the tumor cells themselves. This collaborative evasion renders the tumor resistant to immune surveillance and poses significant challenges for immunotherapy. The pathogenesis of NPC is characterized by a synergistic dialogue orchestrated across multiple axes, including inflammatory pathway co-activation and collaborative immune evasion (59, 60). This synergistic microenvironment, which simultaneously promotes viral replication via the SCFA paradox and shields the tumor from clearance via TIGIT-mediated suppression, is comprehensively summarized in **Figure 3**.

### 3.4 Therapeutic implications: from mechanisms to intervention

Translating these mechanistic insights into clinical practice represents the next frontier in NPC management. Beyond mere description, the potential to manipulate the microbiome for therapeutic benefit is gaining traction. We summarize emerging therapeutic strategies targeting the microbiome-EBV axis—ranging from probiotic restoration to metabolic modulation—in **Table 3**. The clinical outcomes of probiotic interventions, such as those reported by Xia et al. (2019) in **Table 3**, must be critically reconciled with the ‘SCFA Paradox’ discussed in Section 3.2. While systemic administration of probiotics aims to bolster anti-tumor immunity via the gut-nasopharynx axis, it remains unclear whether these benefits are counteracted by the potential of probiotic-derived SCFAs to trigger localized EBV reactivation. This necessitates a more nuanced evaluation of probiotic strains in NPC therapy, moving beyond systemic immune metrics to include local viral kinetics.

**Table 3:** Potential therapeutic strategies targeting the microbiome-EBV axis in nasopharyngeal carcinoma.

Study/clinical trial	Intervention type	Key findings/proposed mechanism	Current status
Xia et al. (2021) (27)	Probiotic Consortium (*Lactobacillus* & *Bifidobacterium*)	Restoration of Mucosal Barrier: Reduced radiation-induced mucositis and modulated immune responses by replenishing beneficial commensals.	Phase II Clinical Trial (Completed)
Microbiome-IO Combination (61)	Combinatorial Immunotherapy (e.g., PD-1 blockade + Probiotics)	Gut-Nasopharynx Axis: Hypothesis that a healthy gut microbiome enhances the efficacy of EBV-specific CTLs and checkpoint inhibitors.	Pre-clinical/Conceptual
Targeting *F. nucleatum* (8)	Selective Antimicrobial Therapy	Disrupting the Synergism: Reducing the load of *F. nucleatum* to dampen NF-κB/LMP1 signaling and halt the “feed-forward” inflammatory loop.	Theoretical Proposal
Epigenetic/Metabolic Modulation (13)	SCFA Mimetics/HDAC Inhibitors	Context-Specific Strategy: Leveraging the “Paradox”—inducing lytic cytotoxicity in B-cells while promoting growth arrest in tumor epithelia.	Experimental Investigation

Probiotic effects in NPC should be interpreted considering the potential site-specific ‘SCFA paradox’ (see Section 3.2).

## 4 Clinical implications: opportunities and cautions

The clarification of the synergistic relationship between the nasopharyngeal microbiome and EBV opens new avenues for clinical intervention, transforming the microbiome from a subject of biological curiosity into a tangible target for diagnosis and therapy.

### 4.1 Diagnostic synergies: integrating viral and microbial markers

Current screening programs for NPC rely heavily on quantifying plasma EBV DNA. While highly sensitive, this method sometimes lacks specificity, distinguishing poorly between transient EBV reactivation and true malignancy. The “microbiome-EBV” synergy suggests a combinatorial approach.

- Composite Biomarkers: A multi-marker panel combining plasma EBV DNA with the abundance of specific nasopharyngeal pathogens (e.g., F. nucleatum, *Porphyromonas*) could significantly enhance diagnostic accuracy. Since microbial dysbiosis often correlates with tumor burden and EBV load, a “Microbial-Viral Risk Score” could better stratify high-risk populations in endemic regions. We propose integrating a quantitative “F/L Ratio” (the relative abundance of *Fusobacterium nucleatum* to *Lactobacillus* spp.) into existing screening models. This ratio could serve as a sensitive bio-indicator, potentially distinguishing between the transient viral fluctuations seen in healthy carriers and the sustained, dysbiosis-driven inflammation characteristic of early-stage carcinoma (7, 15). While the Fusobacterium-to-Lactobacillus (F/L) ratio serves as a potent diagnostic anchor, it should be integrated into a broader ‘Microbial Risk Score’ to capture the full complexity of the NPC-associated niche (8). In this composite model, the F/L ratio represents the balance between primary ‘driver’ pathogens and protective commensals (62). However, to increase sensitivity, the absolute abundances of other key taxa identified in longitudinal studies—specifically *Porphyromonas gingivalis* and *Prevotella* spp.—should be weighted alongside this ratio (9). We propose a tiered diagnostic approach where the F/L ratio acts as the primary screening threshold; cases exceeding this threshold are further stratified by the presence of *Porphyromonas* and *Prevotella*, which are often co-localized with tumor cells and correlate with higher EBV DNA loads. This multi-taxa integration reflects the transversal behavior of the dysbiotic layer rather than a single-species association (63). Such a non-invasive ‘Microbial Biopsy’ could be particularly valuable for stratifying patients with fluctuating EBV DNA loads who lack visible nasopharyngeal lesions.
- Clinical Utility: This ratio could serve as a sensitive bio-indicator to distinguish between the transient viral fluctuations seen in healthy carriers and the sustained, dysbiosis-driven inflammation characteristic of early-stage carcinoma (7, 15). Crucially, this “Microbial Biopsy” would be particularly valuable for stratifying patients with fluctuating EBV DNA loads who lack visible nasopharyngeal lesions on endoscopy, offering a molecular tool to guide closer surveillance or early biopsy (64).
- Early Detection: Given that microbial shifts may precede extensive tumor invasion, monitoring the nasopharyngeal microbiota could potentially serve as an early warning system for individuals with fluctuating EBV titers who have not yet developed clinically detectable lesions (64).

### 4.2 Therapeutic strategies: balancing immunity and viral reactivation

Manipulating the microbiome offers a promising adjunct to standard chemoradiotherapy, but the complexity of EBV biology mandates a cautious approach.

- Probiotics and Fecal Microbiota Transplantation (FMT): Restoring the depletion of commensals like *Lactobacillus* and *Bifidobacterium* could theoretically enhance mucosal barrier function and reduce inflammation. In the context of immunotherapy (e.g., PD-1 blockade), optimizing the gut-nasopharyngeal axis via FMT might improve T-cell responses, similar to successes seen in melanoma (65, 66).
- The Therapeutic Caution (The SCFA Risk): A critical caveat arises from the “SCFA Paradox” discussed in Section 3.2. Indiscriminate administration of probiotics or prebiotics that elevate systemic or local butyrate levels carries a theoretical risk. If these metabolites reach the lymphoid tissue of the nasopharynx, they could inadvertently trigger EBV lytic reactivation in resting B-cells, potentially increasing viral loads and seeding new infections. Therefore, microbiome-based therapies in NPC patients must be carefully tailored to avoid exacerbating viral replication (44, 67).
- Precision Antibiosis and Vaccines: Rather than broad ecological shifts, a safer strategy might involve precision targeting. Bacteriophage therapy designed to specifically lyse F. nucleatum, or vaccines targeting virulence factors like FadA, could dismantle the pro-tumorigenic bacterial scaffold without disturbing the broader commensal community or triggering metabolic risks. This precision is paramount. Unlike broad-spectrum antibiotics, which indiscriminately decimate the commensal microbiome—potentially exacerbating dysbiosis and facilitating secondary fungal infections—lytic bacteriophages offer genus- or even strain-level specificity. This “sniper” approach allows for the eradication of F. nucleatum or *Porphyromonas* while preserving the beneficial *Lactobacillus* populations essential for maintaining mucosal homeostasis and colonization resistance. Furthermore, engineered phages could theoretically be utilized as vectors to deliver genetic payloads (e.g., CRISPR-Cas systems) directly to the pathogenic bacteria, reversing their virulence without killing them, thereby minimizing the release of pro-inflammatory endotoxins caused by bacterial lysis (16, 17, 31).

## 5 Future directions and conclusion

### 5.1 From correlation to causality: the need for NPC-specific models

To move beyond descriptive correlations and establish a causal link between microbial dysbiosis and EBV-driven oncogenesis, sophisticated experimental platforms are required. In the context of personalized medicine, these models serve as functional “patient avatars” for deconstructing individualized microbiome-EBV interactions — moving beyond simple patient characterization (which can be achieved through sequencing) to enable functional drug screening.

**Figure 4:**
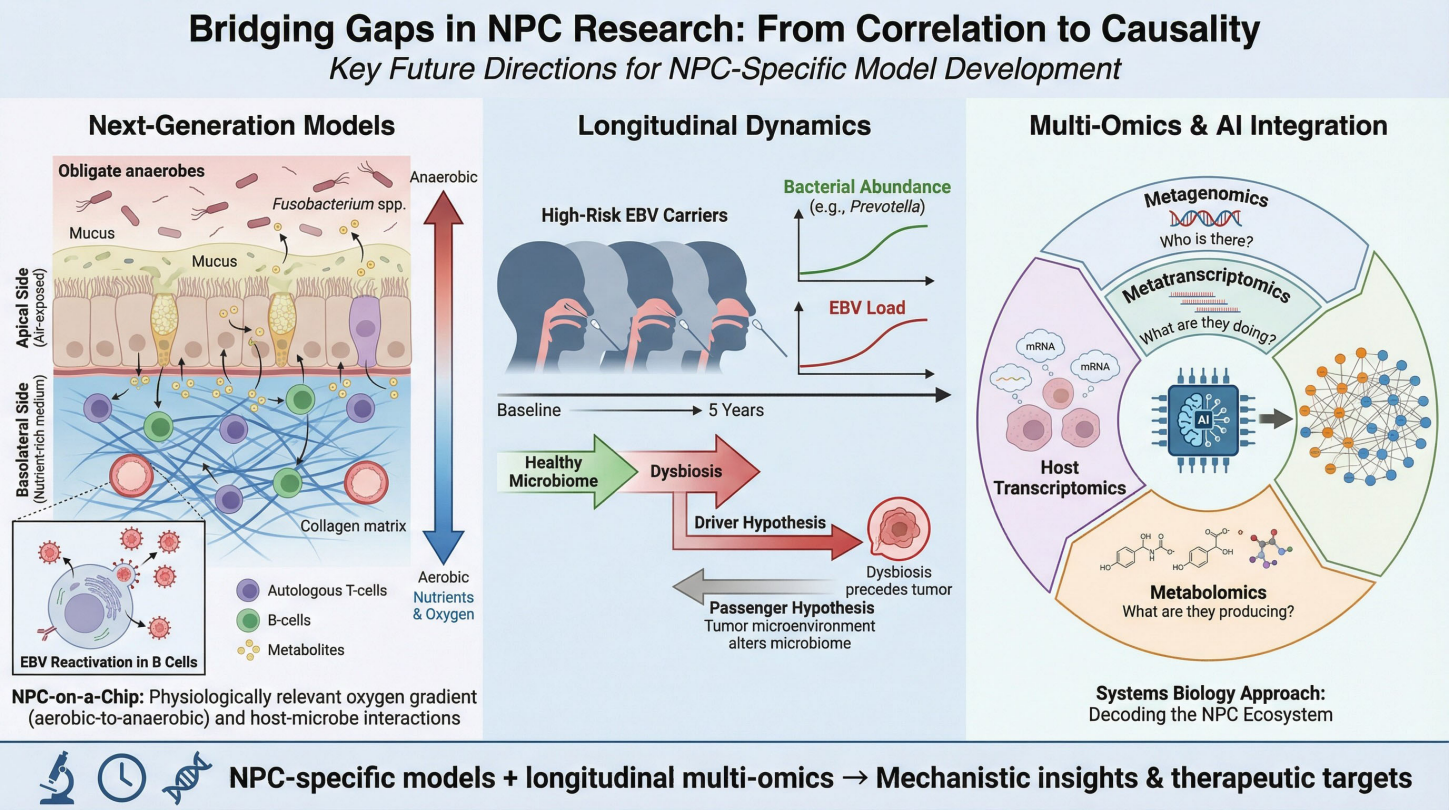
Bridging gaps in NPC research from correlation to causality: key future directions for NPC-specific model development . The Next-Generation Models section (left) highlights the need for ALI and NPC-on-a-Chip platforms. Longitudinal Dynamics (center) emphasizes resolving the Driver vs. Passenger hypothesis over time. Multi-Omics & AI Integration (right) illustrates the systems biology approach required to decode the NPC ecosystem.

- Next-Generation Models: To accurately recapitulate the aerobic-anaerobic transition zone, simple submerged cell cultures are insufficient. The field must adopt 3D organoid systems maintained at an Air-Liquid Interface (ALI). In these models, basal epithelial cells differentiate into a pseudostratified layer containing functional cilia and goblet cells, exposed to air on the apical side while receiving nutrients from the basolateral side. This setup is the only way to physiologically model the oxygen gradient required for the co-culture of obligate anaerobes (apically) and host cells For personalized medicine, this provides an essential platform to functionally validate how specific patient-derived metabolites or precision therapies (e.g., bacteriophages) interact with the host-virus axis. By integrating autologous immune cells (T cells and B cells) into the sub-epithelial collagen matrix of these ALI organoids, researchers can construct an “NPC-on-a-chip” platform. This allows for the real-time visualization of how specific bacterial metabolites penetrate the epithelium to trigger viral reactivation in the underlying B-cell compartment (68–70).
- Longitudinal Dynamics: Future clinical studies must shift from cross-sectional snapshots to longitudinal designs. Tracking the nasopharyngeal microbiome in high-risk EBV carriers over time is the only way to determine whether dysbiosis is a “driver” that precedes tumorigenesis or a “passenger” resulting from the tumor microenvironment (71).
- Multi-Omics and AI Integration: Finally, the complexity of these interactions necessitates an integrated analytical approach. Future studies should move beyond simple 16S rRNA gene sequencing. By combining metagenomics (who is there), metatranscriptomics (what are they doing), and metabolomics (what are they producing) with host transcriptomics, researchers can map the global network of the NPC ecosystem. Artificial Intelligence (AI) and machine learning algorithms will be indispensable tools in this endeavor, capable of deciphering non-linear interactions and identifying robust “microbial signatures” that predict therapeutic response or disease recurrence with high precision (72, 73). AI and machine learning algorithms will be indispensable tools in this endeavor, capable of deciphering non-linear interactions and identifying robust microbial signatures; these crucial translational strategies and future model platforms are detailed in **Figure 4**.

### 5.2 Concluding remarks

The pathogenesis of Nasopharyngeal Carcinoma is no longer viewed solely through the lens of viral infection. It is an ecosystemic disease driven by the collision of a pervasive virus (EBV) and a dysbiotic microbiome within a susceptible host. This review highlights that bacteria are not merely bystanders but active collaborators that fuel inflammation, trigger viral reactivation, and shield the tumor from immune attack. Recognizing this synergy challenges the traditional EBV-centric view and underscores the necessity of a holistic therapeutic approach—one that targets not just the virus or the tumor cell, but the microbial partners that empower them. As we unravel the complexities of this cross-kingdom dialogue, we inch closer to a future where nasopharyngeal microbiome modulation becomes a standard pillar of precision medicine for NPC.
